# Single-cell RNA transcriptomic analysis identifies Creb5 and CD11b-DCs as regulator of asthma exacerbations

**DOI:** 10.1038/s41385-022-00556-1

**Published:** 2022-08-29

**Authors:** Xiaojie Liu, Keilah G. Netto, Leon A. Sokulsky, Lujia Zhou, Huisha Xu, Chi Liu, Ming Wang, Huaqi Wang, Hui Li, Guojun Zhang, Paul S. Foster, Fuguang Li, Ming Yang

**Affiliations:** 1 Academy of Medical Sciences & Department of Immunology, College of Basic Medical Sciences, Zhengzhou University, Zhengzhou, Henan 450052 China; 2 Priority Research Centre for Healthy Lungs, School of Biomedical Sciences & Pharmacy, College of Health, Medicine and Wellbeing and Hunter Medical Research Institute, University of Newcastle, Callaghan, NSW 2300 Australia; 3 Department of Physiology, School of Basic Medicine Science, Central South University, Changsha, Hunan China; 4 Medical Research Centre, The First Affiliated Hospital of Zhengzhou University, Zhengzhou, Henan 450052 China; 5 Department of Pulmonary and Critical Care Medicine, The First Affiliated Hospital of Zhengzhou University, Zhengzhou, Henan 450052 China

## Abstract

Immune responses that result in asthma exacerbation are associated with allergen or viral exposure. Identification of common immune factors will be beneficial for the development of uniformed targeted therapy. We employed a House Dust Mite (HDM) mouse model of asthma and challenged allergic HDM mice with allergens (HDM, cockroach extract (CRE)) or respiratory syncytial virus (RSV). Purified lung immune cells underwent high-dimensional single-cell RNA deep sequencing (scRNA-seq) to generate an RNA transcriptome. Gene silencing with siRNA was employed to confirm the efficacy of scRNA-seq analysis. scRNA-seq UMAP analysis portrayed an array of cell markers within individual immune clusters. SCENIC R analysis showed an increase in regulon number and activity in CD11b^-^ DC cells. Analysis of conserved regulon factors further identified Creb5 as a shared regulon between the exacerbation groups. Creb5 siRNAs attenuated HDM, CRE or RSV-induced asthma exacerbation. scRNA-seq multidimensional analysis of immune clusters identified gene pathways that were conserved between the exacerbation groups. We propose that these analyses provide a strong framework that could be used to identify specific therapeutic targets in multifaceted pathologies.

## Introduction

Patients with asthma may sporadically suffer from acute bouts of exacerbations, characterized by heightened airway inflammation and a decline in lung function that could result in hospitalization^1^. Despite recent advances in the development of asthma therapeutics^2,3^, asthma exacerbations represent a significant clinical challenge given the varying responses to treatment exhibited within the asthmatic population^1,4^. Asthma exacerbations represent an episode of heightened lung inflammation superimposed upon a preexisting allergic airway disease^5,6^. Human studies have revealed that exaggerated inflammation in airways is a hallmark feature of asthma exacerbations^7,8^, and both clinical and animal investigations have uncovered that the inflammatory response is usually characterized by the infiltration of both eosinophils and neutrophils in both lung tissues and BAL fluid^9^. Likewise, this is commonly linked to the increased levels of proinflammatory cytokines and chemokines including interleukin (IL)-1β, IL-6, tumor necrosis factor (TNF)-α, and interferon (IFN)-γ, CXCL1, CCL3, and CCL5^10^. These observations are indicative that the aberrant activation of innate immune cells in the lung plays a key role in the pathogenesis of asthma exacerbations. Therefore, an interesting question is if there are shared or unified mechanisms that critically contribute to the pathogenesis of asthma exacerbation induced by different pathogens.

Two common household aeroallergens, house dust mites (HDM) and cockroach (CR), are highly prevalent allergens that can induce allergic disease, in addition to asthma exacerbation in children and adults with asthma^11–14^. Human studies have demonstrated that reduced exposure to HDM or CR at home greatly improves asthma symptoms and decreases hospitalization with severe asthma exacerbations^15,16^. Additionally, respiratory viral infections are considered as the leading causes for asthma exacerbations in children and adults^1,17,18^, with respiratory syncytial virus (RSV), rhinovirus (RV), influenza, and parainfluenza viruses being the most commonly detected viruses during asthma exacerbations^1,17–20^. RSV is a member of the negative-stranded pneumoviridae family and is considered as one of the major causative pathogens for bronchiolitis, wheezing and impaired lung function in children^21^. RSV infection has been demonstrated to cause 7.2% of hospitalization in elderly patients with asthma exacerbation^22^, and RSV-induced airway diseases have been shown to respond poorly to corticosteroid treatment compared to RV exacerbation^23,24^.

Both allergenic and viral asthma-exacerbation triggers induce highly diversified immune responses that contribute to the pathogenesis of asthma exacerbations. For example, RSV initially replicates in airway epithelial cells and activates granulocytes, dendritic cells (DCs), and monocytes/macrophages by the induction of cytokines and chemokines (IL-12, IFN-γ, CCL3, and CXCL10)^25–28^. HDM or CR extract (CRE) exposure, however, results in the increased infiltration of allergic-associated immune cells, characterized by activated mast cells, eosinophil, dendritic cells (DCs), ILC2s, and antigen-specific CD4^+^ Th2 cells^29–31^. Given the varied landscape of cytokines and inflammatory mechanisms between different exacerbations, a better understanding of the detailed information of the immune response in the lung during an asthma exacerbation is urgently required.

In this study, we employed a microfluidic-based system for high-dimensional single-cell RNA deep sequencing (scRNA-seq) to map the RNA transcriptome of CD45^+^ immune cells in the mouse lung of HDM, CRE or RSV-induced asthma exacerbations, as all of the immune cells in lung express CD45 molecule. To profile the complexity of immune cells in the lung, we first establish a mouse model of asthma with HDM sensitization and aeroallergen challenges and then induced exacerbations by re-challenging the diseased mice with HDM, CRE or RSV. A large-scale, high-dimensional analysis of CD45^+^ immune cells purified from the lung was performed, followed by the determination of mapping of major clusters of these cells with scRNA-seq. Signature genes and regulatory networks for these cells were examined in detail. Regulons were then used to examine the intracellular regulation-network in a single cell. The interaction of regulons, including Creb5, JunD, Myc, and Klf16 in DCs and macrophages is consistently expressed in distinct asthma exacerbations, and the inhibition of Creb5 demonstrated that this factor is involved in promoting allergic and viral induced exacerbation.

## Results

### Different triggers induce steroid-resistant airway hyperresponsiveness (AHR) and airway inflammation in a mouse model of asthma exacerbation

**Fig. 1:**
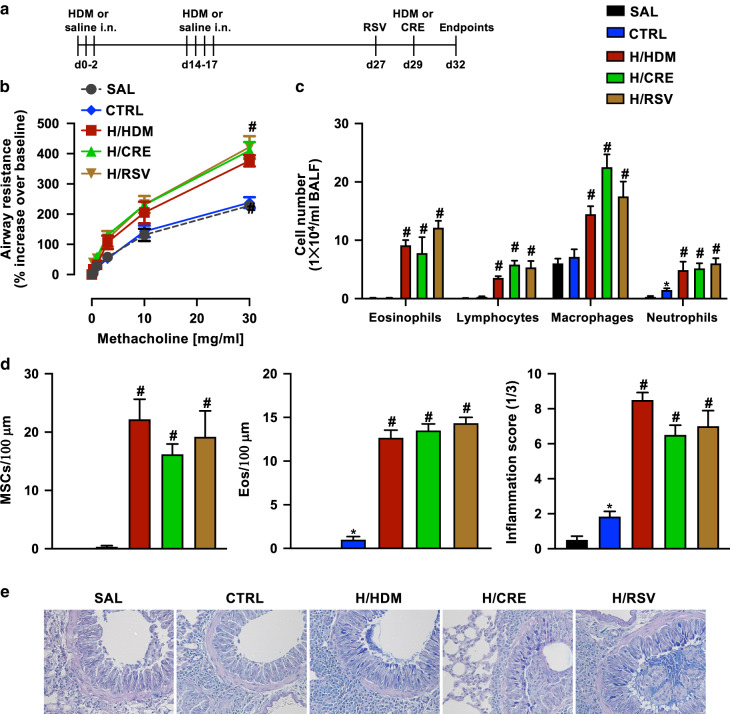
Hallmark features of asthma are upregulated across HDM, CRE, and RSV exacerbated allergic mice. **a** Schematic of in vivo exacerbation models, indicating sensitization phase (days 0–2) to HDM, followed by HDM challenge phase (days 14–17). Exacerbation was induced on day 27 by RSV infection (H/RSV), or on day 29 using allergens (HDM (H/HDM) or CRE (H/CRE)). **b** Measurement of airways resistance determined using flexivent lung-function platform, in response to incremental doses of nebulized methacholine represented as a percentage increase over baseline (PBS only) readings. **c** Differential counts of immune cells isolated from mouse bronchoalveolar lavage fluid (BALF) using light microscopy, represented as cells as per milliliter. **d** Levels of mucus hypersecretion (MSCs) or eosinophil infiltration were assessed and scorings for histopathology (inflammatory infiltrates) were performed on the lung sections. **e** Representative histological pictures of AB-PAS stained mouse lung sections. Values are represented as mean ± SEM, *n* = 6–8. All experiments shown are representative of three independent experiments. #Designates significant differences compared to both SAL and HDM groups (^#^*p* < 0.05); *Designates significant differences compared to SAL group (*p < 0.05). HPF represents high power field.

To investigate the effects of different triggers on the exacerbation of allergic airways disease (AAD), we treated mice i.n with RSV on day 27, or with HDM or CRE on day 29 in the presence of HDM-induced AAD (Fig. 1a). On day 32, lung function and inflammatory responses were examined to establish the AAD phenotype for each group before scRNA sequencing. Both the allergen (H/HDM or H/CRE) groups and the H/RSV groups displayed significantly exacerbated and prolonged AHR when compared to the control group (Fig. 1b). These groups also presented elevated infiltration of eosinophils, lymphocytes, and neutrophils in the BALF (Fig. 1c) in addition to increased mucus hypersecretion, lung inflammation, and worsened lung pathology compared with the control groups (Fig. 1d, e). These features were observed only in HDM sensitized mice and unaffected by dexamethasone treatment (Supplementary Figs. 1 and 2).

### Single-cell RNA sequencing reveals critical immune cell clusters that orchestrate asthma exacerbation in vivo

**Fig. 2:**
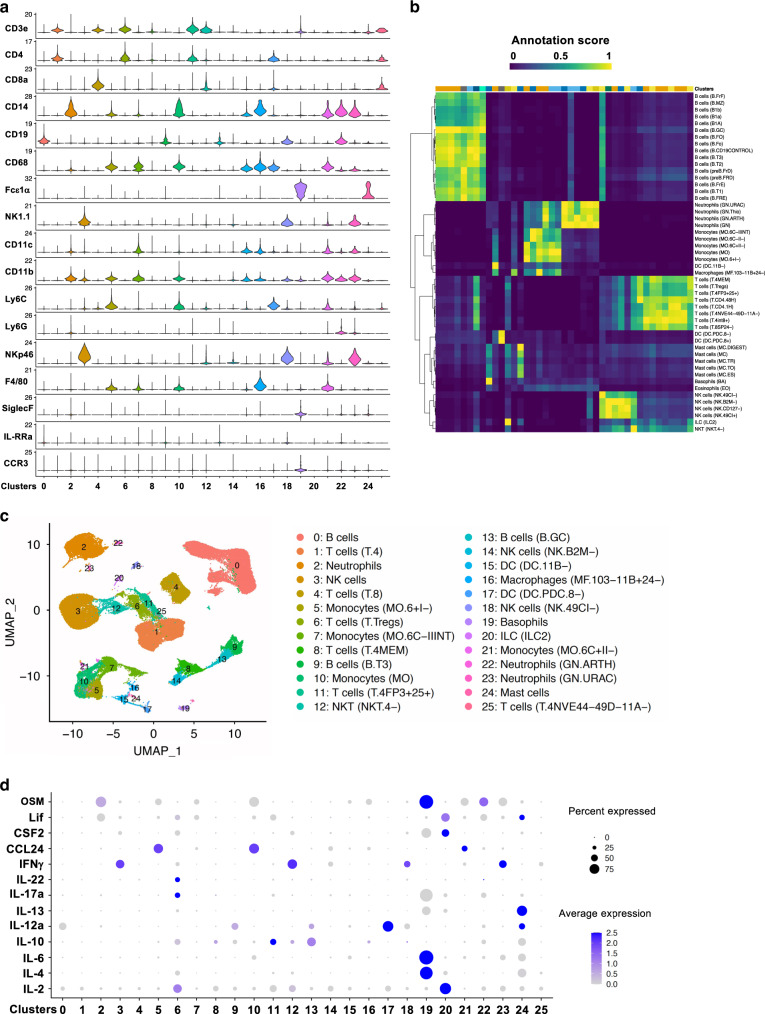
Global map of immune cells in lung revealed by the scRNA-seq analysis in mouse models of asthma exacerbation. Droplet-based single-cell (sc)-RNA sequencing was performed on CD45^+^ single-cell suspensions pooled from six lungs per group including saline, HDM/control (CTRL), HDM/HDM (H/HDM), HDM/CRE (H/CRE), and HDM/RSV (H/RSV) treated animals. All samples were analyzed using canonical correlation analysis with the Seurat R package. Cells were clustered using a graph-based shared nearest-neighbor clustering approach and plotted by a Uniform Manifold Approximation and Projection (UMAP) for Dimension Reduction. **a** Violin plot of expression levels of classical immune cell surface markers found in the CD45^+^ cells in the lung, correlated to relevant immune cluster (clusters 0–25). **b** Heatmap representation of annotation score of immune cell clusters with the expression, by SingleR R package. **c** Graphical representation of 26 major immune clusters identified by scRNA sequencing, represented using UMAP analysis. **d** Dot plot of expression levels of inflammatory factors within identified immune clusters.

To identify immune populations of interest within our exacerbation groups, we first determined the cell clusters of immune cells in lung using Seurat R analysis of scRNA sequencing data. Here, single CD45^+^ immune cell suspensions isolated from exacerbated or control mouse lungs were characterized using Droplet-based scRNA sequencing. Quality control measurements including cell and transcript numbers per sample, distribution of detected genes and gene number per cells and overall complexity (Supplementary Fig. 3), and the distribution of gene and molecule counts (Supplementary Fig. 4) were analyzed. Violin plots were employed to determine the expression of cell surface marker in 26 identified cell clusters by Seurat and SingleR (Fig. 2a). Annotation score by SingleR were presented for each cell cluster (Fig. 2b). For example, T.4NVE44-46G-11A- T cells are naïve CD4^+^CD44-CD46G^-^CD11A^-^ T lymphocytes; Neutrophils (GN.URAC) are CD11b^+^Ly6G^+^ neutrophils stimulated with uric acid; Neutrophils (ARTH) are CD11b^+^Ly6G^+^ neutrophils derived from arthritic mouse; B cells (GC) are splenic CD19^+^IgM^+^IgD^-^GL7^+^PNA^+^ germinal centre B cells. NK cells (NK.B2M-) are splenic CD3^−^ NK cells. The detail of rest immune cell clusters can be found at ImmGen website: www.immgen.org. Furthermore, the distribution of 26 immune clusters was also shown with UMAP (Fig. 2c). The differential expression of signature genes of those cell clusters of five samples was demonstrated in Supplementary Table S1. Among those notable clusters we identified mast cells (C24), basophils (C19), neutrophils (C2), and natural killer cells (C18). Furthermore, we established the inflammatory profile for each immune cluster and presented the 13 most cytokines (Fig. 2d). Here, we found that levels of IL-4, IL-6, and OSM were highly associated with basophils (C19), and IFN-γ was commonly associated with C3, C12, C18, and C23. IL-13, which is highly associated with AHR, was found to be expressed in basophil (C19) populations, and also was strongly associated with mast cells (C24). Eotaxin-2 (CCL24) was found to be secreted from monocyte populations (C5 and C10).

### Differential monocyte myeloid clusters are ubiquitously expressed in RSV, CRE and HDM exacerbated mice, and monocyte cell regulon activity was found to be differentially regulated between allergen and RSV exacerbation asthma groups

**Fig. 3:**
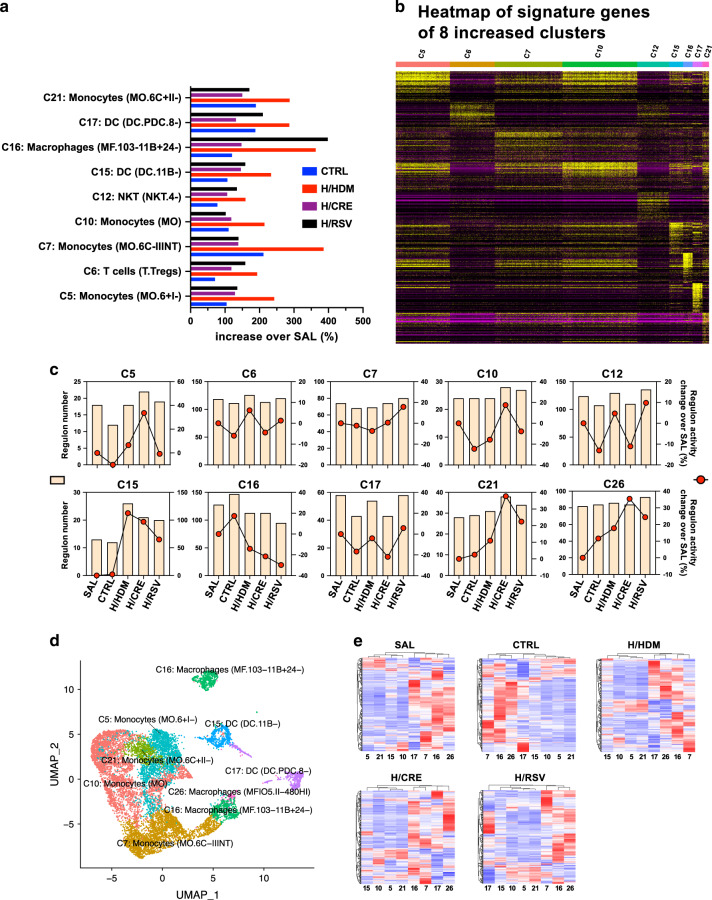
Comparison of immune population clusters between control, allergen, and viral exacerbated mice. **a** Myeloid, Natural killer T cells, and T-regulatory cell populations of each exacerbation group expressed as a percentage increase of SAL cell population levels, as determined by both their proportions among UMAP clustering and cell numbers in the lung (% of UMAP times cell numbers/mL of lung cells). **b** Heatmap of signature genes of clusters C5, C6, C7, C10, C12, C15, C16, C17, and C21. **c** Comparison of both regulon number and the percentage increase of accumulated regulon activity from SAL in myeloid cluster populations between control and exacerbation groups. **d**, **e** Clustered UMAP and heatmap analysis reveal the regulon activation spectra in myeloid immune cell clusters as presented by control and exacerbation groups.

Extending from our preliminary analysis, we then focused on specific groups of clusters between exacerbation groups to determine immune cell populations in allergen/virus exacerbation groups. Here, we found that HDM exacerbated mice had a higher expression of almost all monocyte populations, including monocytes (C5, 10, 21) and dendritic cells (C15 and C17) immune clusters, in addition to Treg cell (C6) and NK cell (C12) immune cluster (Fig. 3a–c). The relative activity of those regulons and the levels of signature genes of these myeloid cell clusters in the five samples were shown in Supplementary Tables S1 and S2. The exception to this was the macrophage cluster (C16), which we found to be expressed the highest in RSV exacerbated mice. CRE exacerbated mice, despite presenting with increased inflammation and AHR, generally had fewer monocyte cells compared to the allergic-only group (HDM), but presented with moderate amounts of Treg cells, macrophages, and DCs. As there were two subpopulations of macrophage (C16), we re-clustered the 8 cell clusters determined previously. We confirmed the previous classification, however, there was an extra small cell cluster, Macrophages (C26), separated from original macrophage (C16). C16 was annotated as MF.103-11B + 24− macrophages that are MHCII^+^ CD11c^+^CD11b^hi^CD103^-^CD24^-^ macrophages purified from mouse lung and C26 as MF.IO5.II-480HI macrophages that are Thio-elicited F4/80^hi^CD115^+^MHCII^-^ macrophages purified from mouse peritoneal cavity, based on ImmGen classification.

Using SCENIC R data analysis, we further investigated monocyte immune clusters within our scRNA-seq dataset in terms of regulon activity. Here, cis-regulatory sequencing was used in conjunction with scRNA-seq data to determine individual regulon activity scores within control and exacerbation groups. Comparison of regulon number and regulon activity within these myeloid clusters were found to be generally variable when compared to SAL mice, allergic mice, and exacerbation mice (Fig. 3d, e). The exception to this was CD11b^-^ dendritic cells (C15), where both regulon number and activity were found to be exclusively high in the exacerbation groups and low in the control samples. Similarly, macrophages (C26) of exacerbation groups were also found to have increased regulon activity and number when compared to that of SAL and CTRL groups. However, the regulon activity of macrophages (C26) in CTRL group was higher than that in SAL group. In addition, the number of regulon of macrophages (C26) was little changed across the five groups. One cluster of monocytes (C21) was found to have a highly upregulated regulon number and activity exclusively in CRE exacerbated mice.

### Pathway analysis of scRNA sequenced exacerbation groups reveal conserved inflammatory profiles in CD11b^−^ DCs involved in the general exacerbation response

**Fig. 4:**
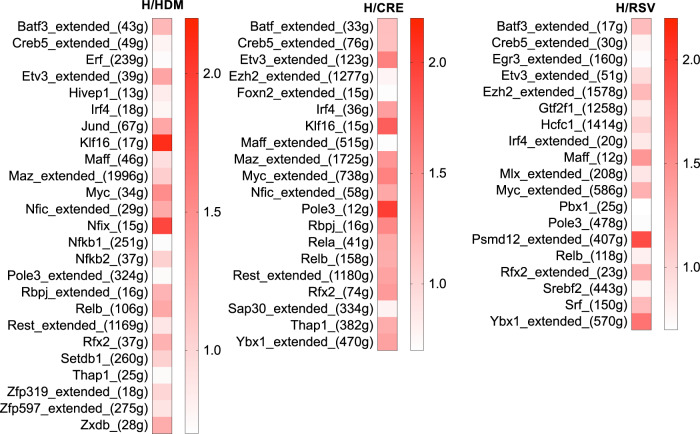
Differential regulon activity in CD11b^−^ DCs (C15) between control and exacerbation groups. SCENIC R package was employed to link the cis-regulatory sequence together with scRNA-seq data and evaluated regulon activity scores for each single-cell reading. Heatmap representation of regulons’ activation, which was increased in exacerbation groups but not in SAL and CTRL groups.

As to what regulons were found to be most commonly activated across CD11b^−^ DCs population (C15) in different exacerbation groups, we explored the most common regulons that were expressed in each exacerbation group that had no or little activity in SAL or allergic-only mice (Fig. 4). Here, we determined that both BAFT3 and ETV3 were consistently upregulated among all three exacerbation groups, indicative that these regulons are involved in driving general exacerbation responses in CD11b^−^ DCs (C15).

To demonstrate the presence of overlapping exacerbation pathways within each exacerbation group, we mapped interactions between identified regulons from our scRNA sequencing data using GeneMANIA (Supplementary Fig. 5A). All three exacerbation groups were shown to have both common regulon factors (shown in pink), group-specific regulon interactions (dark blue in H/HDM, light blue in H/CRE, and green in H/RSV), and in silico predicted regulon factors (grey). Additionally, the presence of hexagon-shaped regulons (e.g., Nfkbie and Nfkb2 in the H/CRE group) indicates the presence of a novel regulome interaction according to the regulomic data. Furthermore, shared regulons between the exacerbation groups primarily constituted of Maff, Etv3, Myc, Irf4, Nfkbie, and Creb5 (Supplementary Fig. 5A). The association of regulons and their molecular targets was demonstrated in Supplementary Table S3. Finally, to determine the significance of conserved regulons between all three exacerbation groups, we utilized ClueGO to demonstrate what shared signaling pathways employed by 166 overlapped genes are activated between the exacerbation groups (Supplementary Fig. 5B and Supplementary Table S4). As shown in this analysis, the most prominent pathways identified include miR-192a and MVP regulation of cell cycle arrest, PIP synthesis, and downregulation of the SMAD2/3:SMAD4 transcription. Most interesting was the existence of notable regulon activation pathways, including ErbB signaling, TNF-α-driven NF-kB activation, and p38 MAPK signaling. Together, these data outline the existence of conserved pathways between discernable asthma exacerbation groups.

### The common-associated regulon factor identified by scRNA sequencing, Creb5, is shown to be associated with macrophage and DC populations in allergic and viral exacerbated mice

**Fig. 5:**
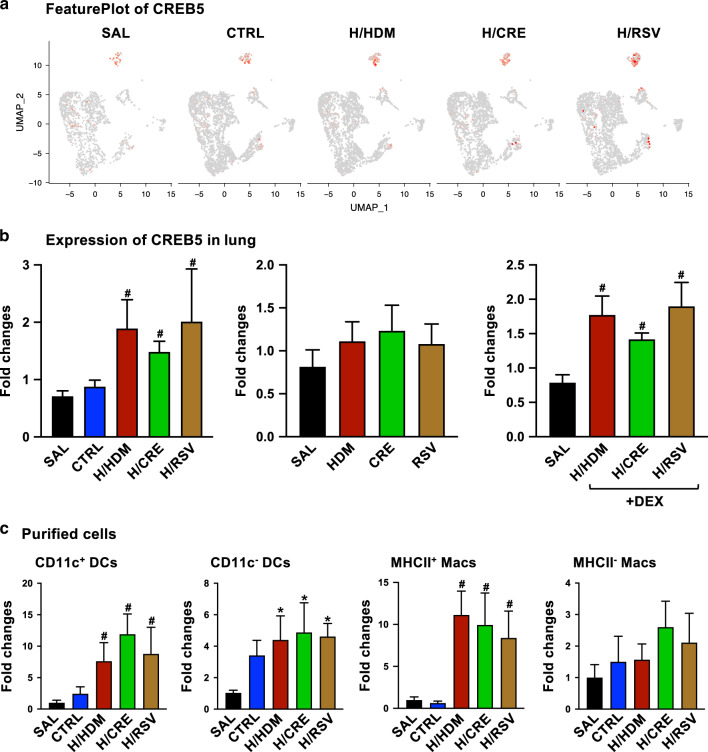
Expression of Creb5 in exacerbation lung, macrophage, and DC populations. **a** Seurat-UMAP cluster analysis showing displaying Creb5 populations between control (SAL and CTRL) and exacerbation (H/HDM, H/CRE, and H/RSV) groups. **b** Fold-change expression of Creb5 determined by qPCR analysis in mouse lungs relative to HPRT. Values are represented as mean ± SEM, *n* = 6–8. **c** Fold-change expression of Creb5 in purified cell populations isolated by factors (±CD11c DCs, ±MHCII macrophages) relative to HPRT. Values are represented as mean ± SEM, *n* = 4. #Designates significant differences compared to both SAL and HDM groups (^#^*p* < 0.05). *Designates significant differences compared to SAL group (*p < 0.05).

The exact relevance of our scRNA-seq findings suggests that conserved regulon factors upregulated in exacerbation groups can provide potential therapeutic targets that can be exploited for treatment. As shown in Fig. 4, the factor Creb5 was a factor that was found to be consistently upregulated between exacerbation groups and was furthermore found to be upregulated both in macrophage (C16) and CD11b^−^DC (C15) populations by UMAP (Fig. 5a). Furthermore, we investigated the expression of Creb5 in whole lung samples from exacerbated mice and confirmed that it was significantly upregulated in HDM, CRE, and RSV exacerbation mice irrespective of Dexamethasone treatment, but not in allergic-only mice and mice treated with HDM, CRE, and RSV only (Fig. 5b). Confirming the expression of Creb5 in DC and macrophage populations, we determined that Creb5 was significantly upregulated in CD11c^+^ DCs (F4/80^lo^CD11b^lo^CD11c^+^MHCII^+^) and MHCII^+^ macrophages (F4/80^hi^CD11b^lo^CD11c^-^MHCII^+^) that have been isolated from allergen or viral exacerbated mice when compared to allergic-only mice (Fig. 5c).

### Depletion of Creb5 results in a marked reduction in AHR, inflammation, and mucus hypersecretion consistently between all exacerbation groups

**Fig. 6:**
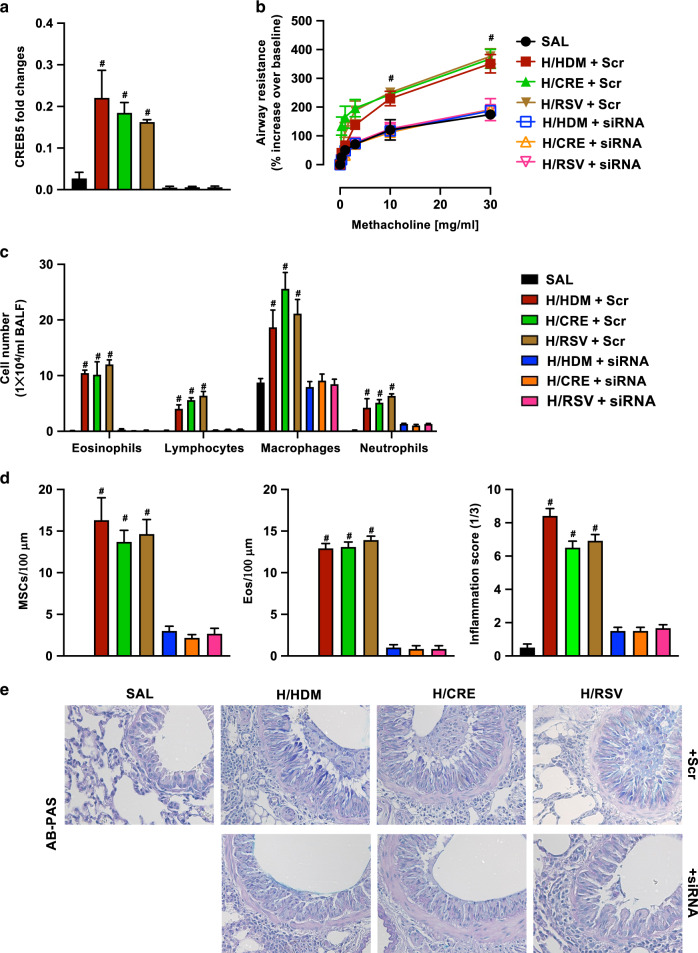
Silencing of Creb5 in exacerbation models results in a profound decrease in inflammation, AHR, and mucus hypersecretion. **a** Fold-change expression of Creb5 determined by qPCR analysis in mouse lungs relative to HPRT. **b** Measurement of airways resistance determined using flexivent lung-function platform, in response to incremental doses of nebulized methacholine represented as a percentage increase over baseline (PBS only) readings. **c** Differential counts of immune cells isolated from mouse bronchoalveolar lavage fluid (BALF) using light microscopy, represented as cells per milliliter. **d** Levels of mucus hypersecretion (MSCs) or eosinophil infiltration were assessed and scorings for histopathology (inflammatory infiltrates) were performed on the lung sections. **e** Representative histological pictures of AB-PAS stained mouse lung sections. Values are represented as mean ± SEM, *n* = 4–6. #Designates significant differences compared to both SAL and siRNA treated groups (^#^*p* < 0.05). HPF represents high power field.

To signify the value of our scRNA sequencing results, we used siRNA to target Creb5 in exacerbation mouse models to validate if scRNA sequencing of regulon regions can act as a viable mechanism to identify viable therapeutic targets. As Creb5 was found to be upregulated in mouse whole lungs, we administered siRNA targeting Creb5 intranasally in RSV, HDM and CRE exacerbated mice and confirmed Creb5 knockdown using qPCR (Fig. 6a). Remarkably, Creb5 knockdown resulted in a significant reduction in AHR irrespective of exacerbation trigger (Fig. 6b), and analysis of isolated BALF showed a marked decrease in lymphocyte, macrophage, neutrophil, and eosinophil populations (Fig. 6c). The histological analysis found that inhibiting Creb5 resulted in a profound decrease in mucus-secreting cells and lung eosinophilia, and presented with a significantly reduced inflammatory score (Fig. 6d, e). Finally, decrease of mucus-secreting cells by Creb5 siRNA was accompanied by significant decreases of Muc5AC, Muc5B and Muc4 RNA as compared to scramble controls (Supplementary Fig. 6)

## Discussion

The clinical challenge of treating asthma exacerbations is in part due to the heterogeneous nature of disease manifestation and etiology. Despite the effectiveness of mainstay therapies in the majority of mild and medium asthmatics, the treatment of severe asthmatics is compromised due to the case-by-case assessments required in determining treatment, and the varying responses exhibited by the patients as a result of a range of allergen or viral triggers. Our use of in vivo modeling of asthma in AAD mice supplemented with either HDM, CRE, or RSV infection, and the use of scRNA deep sequencing of lung immune cells is indicative of the existence of conserved pathways that a central to the manifestation of asthma exacerbation, which could be beneficial in establishing therapeutic targets that can suppress exacerbations in a wider range of asthmatics. Furthermore, we identified cell clusters that highlight cell populations and cytokines that are conserved between different exacerbation groups, identified regulons within monocyte clusters that are important in driving the general exacerbation response, and highlighted potential pathways based on regulon expression that is present in both individual and shared exacerbation groups.

Our study employs the mouse model of HDM-induced asthma, which represents sensitization and induction of disease by directly activating innate and adaptive immunological process in the lung. Further the inflammatory milieu represents the underlying chronic inflammation in asthma. Unique to this study is the method by which we established exacerbation in our HDM sensitized and challenged mice, as we model human exacerbation triggered by a pre-sensitized allergen (HDM), a novel introduced allergen (CRE), and viral infection (RSV). By extension, we propose that the groups presented in this study represent factions of asthma exacerbation patients that would be regularly encountered in a clinical setting: that is, patients that are triggered by encountered or non-encountered allergen, and patients experiencing a viral exacerbation. We chose to use RSV to represent viral-induced exacerbations in this study, as opposed to RV or FLU, both due to the prevalence this virus has in inducing asthma exacerbation in children, and the asthmatic phenotype observed in the H/RSV group was comparable to the H/HDM and H/CRE group in terms of AHR, lung inflammation, mucus production and steroid resistance (Supplementary Figs. 1 and 2).

Our single-cell RNA deep sequencing revealed a wealth of myeloid and lymphatic clusters in our exacerbation groups. The most prominent clusters include B cells, T cells, neutrophils, natural killer cells, monocytes, and dendritic cells. While not surprising, given the influx of cells that are commonly associated with asthma exacerbations, the average expression of these clusters was found to be consistent between exacerbation groups for most clusters. Exceptions for this, however, were monocyte cluster populations, including monocytes (C5, C7, C10, and C21), DCs (C15 and C17), NKTs (C12), and macrophages (C16), in addition to Treg cells (C6). Consistent with this was the upregulation of chemotactic factors including CCL24, which was notably upregulated in monocyte populations (C5 and C10), IFN-γ in NK cells (C3) and NKT cells (C12), and IL-12a expression in DC cells (C17). Surprisingly, basophils (C19) were found to be a significant source of IL-4, IL-6, and OSM, indicative that these cells could be significant drivers of exacerbation in these models. Although basophils and mast cells both expressed IL-4 and IL-13 to certain extend in our study, basophils preferably expressed IL-4 and mast cells generated more IL-13. Furthermore, our result has shown that ILC2s expressed both IL-4 and IL-13 albeit at low level, indicating their contribution to type 2 immunity. Interestingly, ILC2s are one of the early sources of IL-4 and IL-13 to respond rapidly to allergic reactions^32^. However, IL-2 rather than IL-4 produced by ILC2s is critical for the maintenance of type 2 immune response^33^, which further validates our observation that ILC2s express greater amount of IL-2. This reflects the complex roles of these immune cells in the interaction between host and pathogens.

As the most varied cluster observed between the exacerbations groups, monocytes were closely examined using SCENIC to determine the regulon activity within each monocyte cluster. Our observations found that there were a profound number of regulons that were actively conserved within these populations (namely Batf3, Creb5, and Etv3). The rationale of investigating population regulon activity is two-fold, as it demonstrated what immune clusters are most activated and what specific immune pathways are activated in these exacerbation groups. Namely, the most activated cluster consisted of CD11b^−^ DCs (C15), as not only the number of regulons but also the regulon activity of this cell cluster was high across the three exacerbation groups. It is likely that CD11b- DCs play a key role in the pathogenesis of asthma exacerbation in general. Although asthma exacerbation patients present with varying levels of leukocytes and lymphocytes, monocytes (particularly local macrophage populations within the lung) are typically present in asthma patients in general, thus it is not surprising that such immune clusters had shared regulon features across the exacerbation groups. As shown by ClueGO analysis, these conserved regulon pathways were found to have an active role in perpetuating downstream inflammatory pathways (FAS, NF-kB, and MAPK signaling), in addition to upstream factors such as TLR4. Surprisingly, the most shared pathway between the three groups was a specific miRNA pathway (miR193a) that regulates the expression of major vault protein (MVP). MVP has previously been reported to be a negative regulator of IKK and NF-kB signaling^34^ and has been shown to regulate the growth and survival of smooth muscle cells in severe asthma^35^. Our scRNA deep sequencing analysis, therefore, provides a basis for exploring this pathway in asthma exacerbation and suggests that miR193a and MVP should be explored in future studies.

As a proof of concept, we focused specifically on the conserved exacerbation factor Creb5 to demonstrate how regulon factors identified by scRNA sequencing can be exploited as a general therapeutic target for asthma exacerbations. Creb5 encodes for Cyclic AMP-response element-binding protein 5, and while this gene has been identified in datasets focused on asthma exacerbation and non-Th2-driven asthma^36,37^, very little focus has been given to its role in asthma exacerbation. Here, Creb5 inhibition through siRNAs resulted in a marked decrease of features associated with asthma exacerbation (inflammation, AHR, and mucus secretion) irrespective of the exacerbation trigger. It should be noted that this inhibition could also be occurring in other cells as siRNA treatment would not be specifically inhibiting Creb5 in CD11b^−^ DCs exclusively in the current study. Nevertheless, the implication of this finding suggests that our scRNA sequencing methodology has the potential to identify critical factors that co-exist between disease phenotypes and could be applicable across a range of heterogeneous diseases.

Interestingly, we were able to separate a small macrophage cluster from original C16 after re-clustering. C16 was annotated as MF.103-11B+24− macrophages that are MHCII^+^CD11c^+^CD11b^hi^CD103^−^CD24^−^ macrophages purified from mouse lung and C26 as MF.IO5.II-480HI macrophages that are Thioglycollate-elicited F4/80^hi^CD115^+^MHCII^−^ macrophages purified from mouse peritoneal cavity, based on ImmGen classification^38^. The former macrophage subpopulation is interstitial macrophages^39^ and the latter one large peritoneal macrophages (LPM)^40^. Under homeostatic condition, LPM can be self-renewal and independent of haematopoiesis^41^. LPM play an important role in the regulation of phagocytosis of apoptotic cell and wound healing and are able to generate high levels of G-CSF, GM-CSF and KC in response to inflammatory stimulus^42^. This evidence suggests a protective role of C26 cell cluster in the lung. However, how these cells migrate into inflamed lung or if they are previously unidentified macrophage subpopulation in lung remain obscured. Future studies of this subpopulation of macrophage in lung may not only provide insight into the detail function of macrophages, but also develop new treatment by promoting the activation of these cells to control lung inflammation.

Together, our study identifies factors within different models of asthma exacerbation that are conserved, both in terms of monocyte population clusters and regulon activity. These analyses are indicative of the existence of shared, pro-exacerbation parties that are not specific for a specific exacerbation trigger, which would have broad implications for developing general therapies for large populations of asthmatic patients. Additionally, future studies may focus on elucidating the specific cell types that employ Creb5 in promoting exacerbating inflammation. It is thus likely that scRNA-seq of distinct asthmatic patient cohorts, using the framework demonstrated in this study, could provide clinical benefits in the pursuit for targeted patient-wide asthmatic therapeutics, and that the regulons identified in this study could be a contributing factor involved in these conserved patient pathways.

## Materials and methods

### Mice

Six to eight weeks old pathogen-free BALB/c mice (male and female) were obtained from the University of Newcastle and Zhengzhou University. All experiments were performed with approval from the local animal ethics committee of the University of Newcastle (#A-2016-616), Australia, and Zhengzhou University (#ZZURIB20180120), China.

### Experimental model

Mice were sensitized with 50 µg of HDM (*Dermatophagoides pteronyssinus*) extract (Greer Laboratories, Lenoir, NC) in 50 µL sterile saline intranasally (i.n) on days 0, 1, and 2. Two weeks later, mice were challenged i.n with 5 µg of HDM for four days (days 14–17). To induce exacerbation, mice were exposed to either CRE (Greer, 5 µg in 50 µL) i.n (H/CRE group) or HDM (5 µg in 50 µL) i.n (H/HDM group) on day 29. In some experiment, mice were stimulated with 0.5 × 10^6^ Plaque-Forming Units (PFU) of RSV per mouse (i.n) on day 27 (H/RSV group). In some experiment, HDM sensitized/challenged mice were exposed to saline i.n on day 29 as exacerbation control (HDM group). Control mice were sensitized and challenged with saline and further exposed to saline i.n on day 29 (SAL group). The endpoints occurred on day 32, mice were anesthetized for lung-function measurement, followed by bronchoalveolar lavage fluid (BALF) and tissue collection for analysis. For siRNA experiments, siRNA targeting Creb5 (5′UUAUGAACAGUAUAGGUUU3′) or NONc siRNA were purchased from Dharmacon (USA) and administered to mice intranasally at 3.75 nmol/50 mL after being anesthetized with isoflurane, on day 26 for the RSV-induced exacerbation group, or day 28 for HDM or CRE-induced exacerbation groups.

### Measurement of lung function

Airway resistance (Raw) was measured using a Flexivent apparatus (FX1 system; Scireq) in response to increasing doses of methacholine (Sigma-Aldrich)^43,44^. Briefly, mice were anesthetized i.p by a mixture containing xylazine (2 mg/mL i.p.; Troy Laboratories), ketamine (40 mg/mL; Parnell), and PBS (1:4:5). A cannula was inserted into the trachea, and mice were ventilated with a tidal volume of 8 mL/kg at a frequency of 450 breaths/minute with a positive expiratory pressure of 2 cm H_2_O. Mice were challenged with saline aerosol, followed by increasing concentrations of methacholine (0.3, 1, 3, 10, and 30 mg/mL) for 15 s at each dose. Measurements were excluded if the coefficient of determination was lower than 95%. Airway resistance was recorded and presented as the percentage increase over baseline.

### Bronchoalveolar lavage fluid

BALF was collected from sacrificed mice, using a technique previously described^45^. Briefly, the left lobe of the mouse lungs was tied off and the right lung was flushed twice with 700 µL Hanks Balanced Salt Solution (HBSS; Invitrogen). BALF samples were centrifuged to yield a pellet. Red blood cells were lysed using 0.86% (w/v) ammonium chloride. Total cell counts were then determined by haemocytometry, and the remaining cells cytospun onto glass slides. Differential leukocyte counts were determined using morphological criteria and light microscopy (×100) on May-Grünwald and Giemsa stained slides, counting 300 cells/slide/sample.

### Lung histology

Lung tissues were fixed in 10% neutral buffered formalin for 24 h before being transferred to 70% ethanol. Lungs were paraffin-embedded and sections stained for eosinophil quantification using Congo Red, or for mucus-secreting cells (MSCs) using an Alcian Blue (AB)-PAS staining. The number of MSCs and eosinophils were determined by morphological criteria around major airways under the light microscopy at ×400 original magnification and quantified as described previously^46^. Scoring for histopathology (inflammatory infiltrates) was performed according to a set of morphological criteria, as previously described^47–49^. All scoring was performed blinded. Histological photos were taken using the Zeiss AXIO imager microscope system.

### RNA extraction, RT-PCR, and quantitative PCR

Total RNA from lung tissue or purified cells were extracted using TRIzol reagent (Invitrogen) as previously described^46^, then quantified using the NanoDrop 1000 spectrophotometer (NanoDrop). The cDNA was synthesized by RT-PCR using random hexamer primers (Invitrogen) on a T100 thermal cycler (Bio-Rad). The quantitative PCR was performed on a Viia7 real-time PCR machine (Life Technologies) using SYBR reagents. The levels of mRNA were normalized to hypoxanthine phosphoribosyltransferase (HPRT, internal control) and expressed as a fold change relative to the control samples (SAL group).

### Lung immune cell purification

Lung immune cells were prepared and pooled for scRNA-seq, using six mice from each group. Mice were sacrificed by i.p. injection of an overdose of pentobarbital. After thoracotomy, the pulmonary circulation was perfused using 37 °C PBS to remove intravascular cells. Lung tissues were mechanically minced using fine scissors into RPMI1640 containing digestion enzymes (3 mg/mL Collagenase IV; Worthington Biochemical) and 40 mg/mL DNase I (Sigma-Aldrich) before being incubated for 30 min at 37 °C and 5% CO_2_ with gentle shaking every 5–10 min. Samples were depleted of erythrocytes using 0.86% (w/v) ammonium chloride. After washing, a single-cell solution with 1 × 10^7^ cells/mL was prepared using a 100 μm filter. Cells were then washed with ice-cold 1% FCS/PBS. Fc receptor blocker (anti-mouse CD16/32, 1:100) was added, followed by anti-mouse CD45 antibody (1:300) and washing with ice-cold 1% FCS/PBS twice. Cells were stained with Fixable Viability Dye 7-AAD (1:40) for the exclusion of dead cells. Lung immune cells were purified with a BD AriaIII, with a high degree of purity (routine over 95%).

### Droplet-based scRNA-seq

Immediately post-sorting, CD45^+^7-AAD^−^ single cells were run on a 10× Chromium system (10× Genomics)^50–52^. A library preparation by Novogene (Beijing, China), following the recommended protocol for the Chromium Single-Cell 30 Reagent Kit (v2 Chemistry). Libraries were run on the HiSeq4000 for Illumina sequencing. Post-processing and quality control were performed using a 10× Cell Ranger package (v1.2.0; 10× Genomics). Reads were aligned to the mm10 reference assembly (v1.2.0; 10× Genomics). Primary assessment with the 10× Cell Ranger for the sample of CTRL group reported 10, 957 cell barcodes with 1791 median genes per cell sequenced to 97.2% sequencing saturation with 98,117mean reads per cell. Primary assessment with this software for the sample of HDM group reported 14,455 cell barcodes with 1655 median genes per cell sequenced to 97.0 % sequencing saturation with 64,046 mean reads per cell. Primary assessment with this software for the sample of H/HDM group reported 12,858 cell barcodes with 1694 median genes per cell sequenced to 97.1% sequencing saturation with 66,129 mean reads per cell. Primary assessment with this software for the sample of H/CRE group reported 14,594 cell barcodes with 1579 median genes per cell sequenced to 97.2% sequencing saturation with 59,460 mean reads per cell. Primary assessment with this software for the sample of H/RSV group reported 14,534 cell-barcodes with 1671 median genes per cell sequenced to 97.4% sequencing saturation with 61,093 mean reads per cell.

### Bioinformatic analysis of scRNA-seq data

Gene expression matrixes generated by the 10× Cell Ranger aggregate option were further analyzed with R package Seurat (version 3.0) with default parameters. The data were filtered according to the following thresholds: less than 200 or greater than 2500 as unique expressed genes (nFeature_RNA) and greater than 5% as the percentage of mitochondrial genome content to remove low-quality readouts. DoubletFinder R package to verify the filtration of doublets in the five datasets individually^53^. The data were then normalized by converting with a scale factor (default as 10,000) and log-transformed with the Seurat embedded function. A correlation analysis was performed by employing the RunPCA function of the Seurat package, following by an integrated analysis of the five datasets which were then integrated with SelectIntegrationFeatures, PrepSCTIntegration, FindIntegrationAnchors, and IntegrateData functions of Seurat sequentially. Clustering analysis was carried out with standard Seurat package procedures, with a resolution at 1.2. The identified clusters were then visualized using Uniform Manifold Approximation and Projection (UMAP) of the principal components in Seurat. Average gene expression matrixes were then retrieved for each cluster and differential expression among clusters was performed to identify the top markers at a high level by each cluster, with the FindAllMarkers implemented function (parameters: only. pos = FALSE, min. pct = 0.2, thresh. use = 0.2). Cell clusters were annotated with SingleR, for unbiased cell type recognition of scRNA-seq, and compared with the Immunological Genome Project (ImmGen; reference mouse dataset)^54^. Spearman correlation analysis was performed on variable genes after comparing with each sample in the reference dataset. The coefficient of multiple correlation (per cell type) was aggregated to provide a single value per cell type per single cell. For some UMAP plots, feature plots were demonstrated for selected marker genes using the Seurat function FeaturePlot.

Pathway enrichment analysis examining enriched processes in clusters was performed using IPA^55^. Gene ontology (GO) enrichment analyses were performed with topGO package in R (Bioconductor)^56^. To uncover the KEGG pathway that is potentially linked to the 319 identified genes in C16 cluster, pathway enrichment analysis was performed by ClueGO plugin of Cytoscape software^57^. A right-side hypergeometric test was employed for calculation of the *P* value, followed by a Benjamini–Hochberg adjustment. A pathway with adjusted *P* value <0.05 was considered significant.

To examine the activity of transcriptional factors (TFs), cis-regulatory analysis using SCENIC R package was employed^58^. It infers the gene regulatory network based on co-expression and DNA motif analysis. The network activity was then analyzed in each cell. TFs were determined using GENIE3 and compiled into modules (regulons) that were further subjected to cis-regulatory motif analysis by RcisTarget with two gene-motif rankings: 10 kb around the Transcription Start Sites (TSS) and 500 bp upstream. Regulon activity in every cell was then scored using AUCell. Finally, a network of the regulons was analyzed via GeneMANIA^59^.

### Statistical analysis

We have employed multiple methods to minimize confounding variables, including a random number table, matching experimental procedures and scoring was performed blindly. Statistical analysis was performed using GraphPad Prism version 8.3 (GraphPad Software, USA). Two-way ANOVA was used to identify differences between two or more experimental groups, and Student’s unpaired *t*-tests were used where comparisons were made between two treatment groups. All results are presented as mean ± SEM, and *P* values of <0.05 were considered statistically significant. Data are available upon request.

## Supplementary infomation

Supplementary tableS1
Supplementary tableS2
Supplementary tableS3
Supplementary tableS4
Supplementary information
