# Supplementary material for: Single-cell RNA transcriptomic analysis identifies Creb5 and CD11b-DCs as regulator of asthma exacerbations
Source: Mucosal Immunol. 2022 Aug 29;15(6):1363–74. doi: 10.1038/s41385-022-00556-1 (PMC9705253; doi:10.1038/s41385-022-00556-1)
Supplement: Supplementary file 5 — Supplementary information [file 41385_2022_556_MOESM5_ESM.pdf]

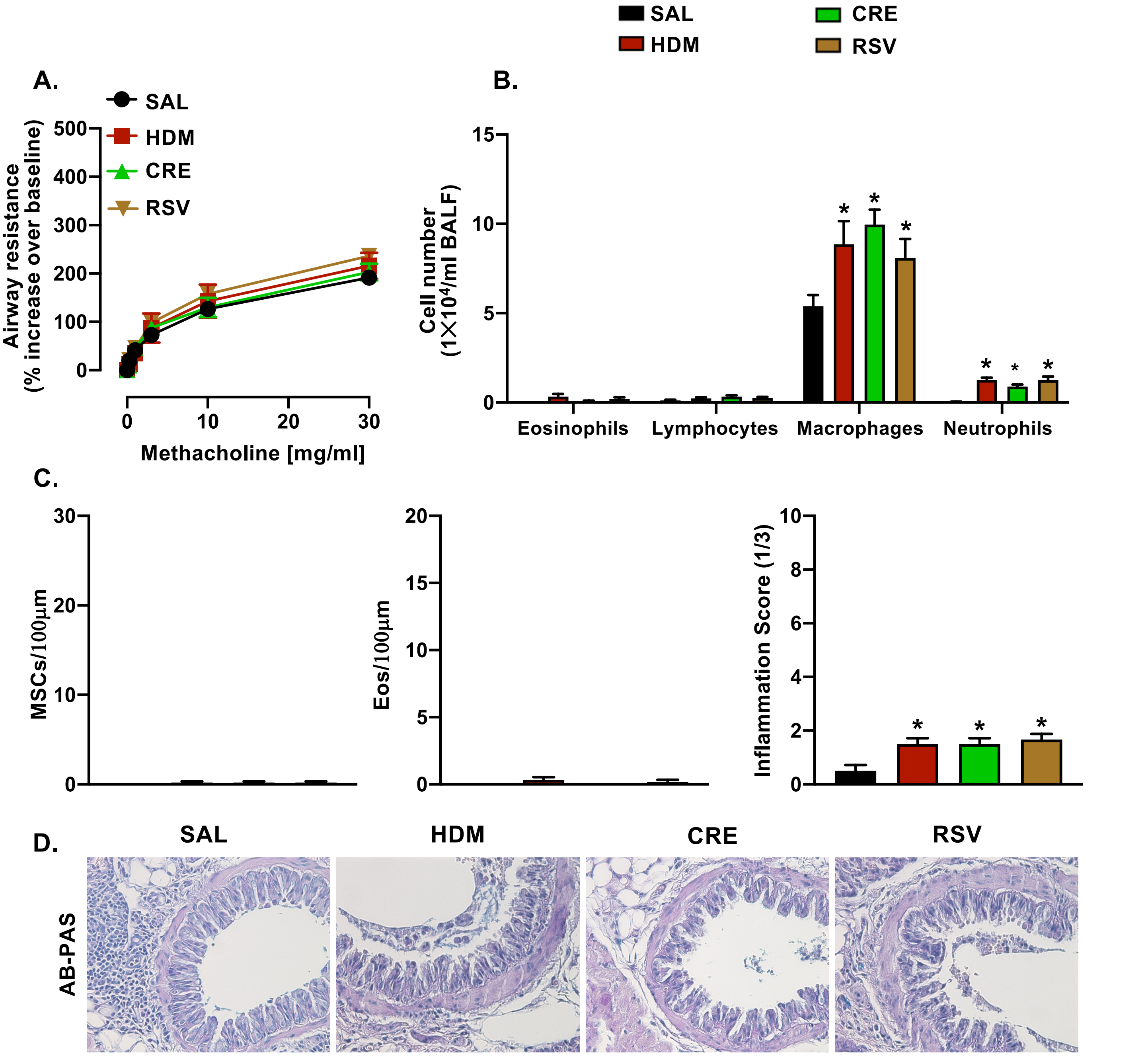

Supplementary Figure 1: Treatment with RSV or a single dose of CRE or HDM does not induce asthma exacerbation features in non-allergic mice. (A) Airways resistance presented as a percentage over baseline in response to increased methacholine doses as determined by flexivent. (B) Cell enumeration from BALF using total cell counts and differential cell counts via light microscopy. (C) Scorings for PAS (mucus secretion) and histopathology (eosinophils and inflammation scores) were performed with the lung section. Lung sections were assessed for mucus hypersecretion (MCSs) or eosinophil infiltration per 100µm high power field (HPF). (D) Representative histological pictures of AB-PAS or Congo Red stained mouse lung sections. Values are represented as mean ± SEM, n=6–8. *Designates significant differences compared to SAL group (* $p < 0.05$).

Fig S1.

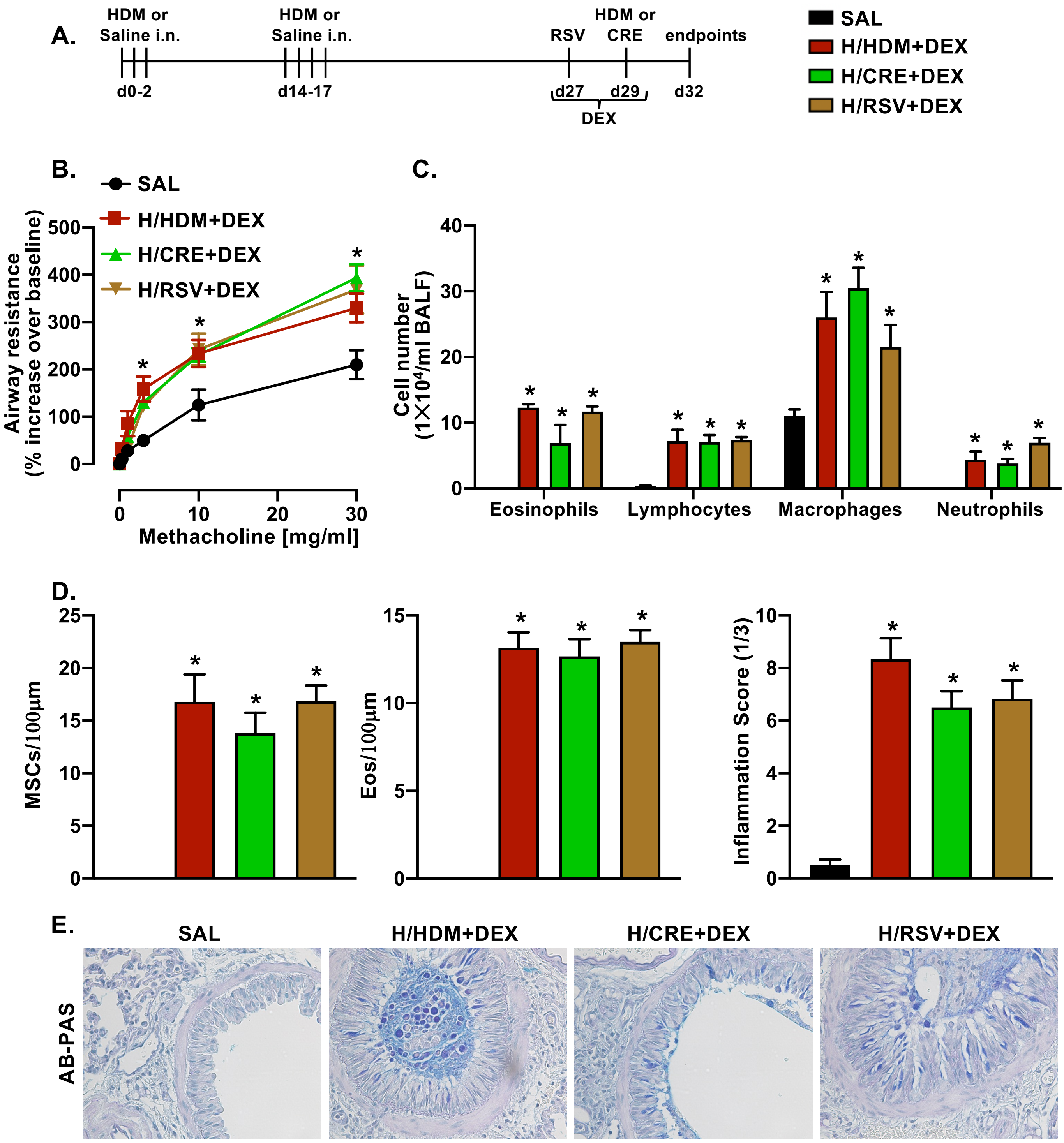

Supplementary Figure 2: RSV infection, HDM or CRE induced AHR, and airway inflammation is glucocorticoid resistant. BALB/c mice were sensitized and exposed to HDM i.n. for the establishment of AAD (saline treatment was used as a control). HDM, CRE, or RSV were administered i.n. for the induction of asthma exacerbation as described in (A) experimental schematic. Dexamethasone (DEX, 1mg/kg) was administered i.p. on days 26, 28, and 30. (B) Airways resistance presented as a percentage over baseline in response to increased methacholine doses as determined by flexivent. (C) Cell enumeration from BALF using total cell counts and differential cell counts via light microscopy. (D) Scorings for PAS (mucus secretion) and histopathology (eosinophils and inflammation scores) were performed with the lung section. Lung sections were assessed for mucus hypersecretion (MCSs) or eosinophil infiltration per 100μm high power field (HPF). (E) Representative histological pictures of AB-PAS or Congo Red stained mouse lung sections. Values are represented as mean ± SEM, n = 6–8. *Designates significant differences compared to SAL group (*$p < 0.05$).

Fig S2.

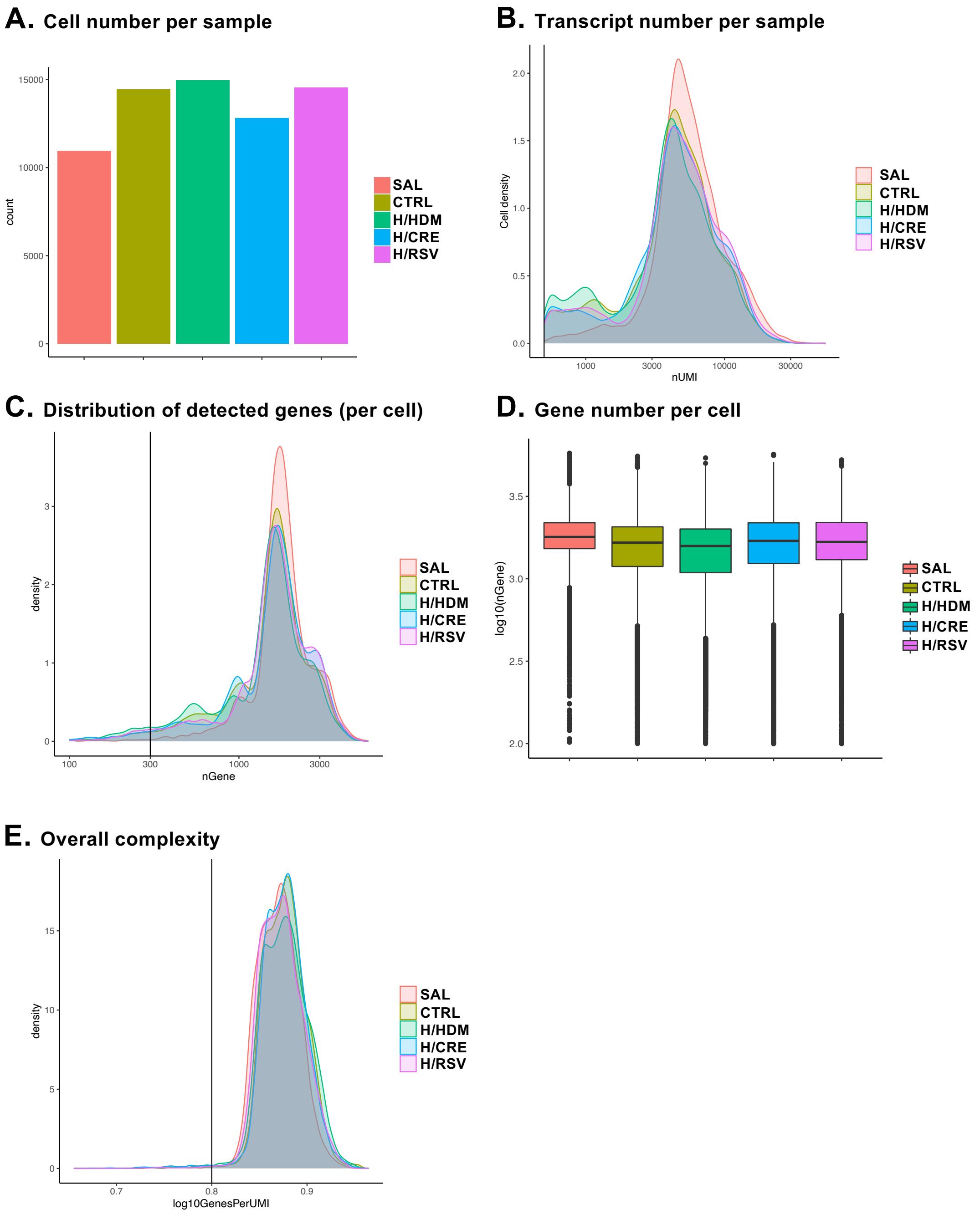

Supplementary Figure 3: Quality control measurement of scRNA sequencing samples. (A) The average number of clustered cells in each experimental group. (B) The average number of detected transcripts per sample in each experimental group is shown using Seurat software. (C-E) Distribution, gene number, and overall complexity of per cell as shown between experimental groups as shown using Seurat software.

Fig S3.

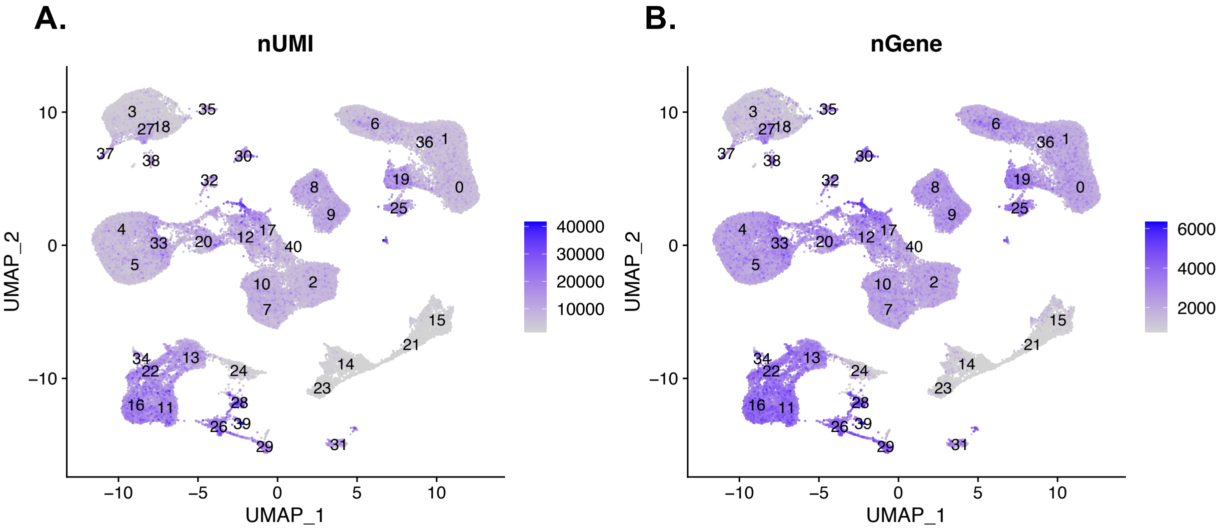

Supplementary Figure 4: Distribution of gene and molecule counts. Single-cell RNA-seq was performed on single-cell suspensions pooled from six lungs per group including saline (SAL), HDM + vehicle (CTRL), HDM+HDM (H/HDM), HDM+CRE (H/CRE), and HDM + RSV (H/RSV) exposed mice. All samples were analyzed using canonical correlation analysis with the Seurat R package. Cells were clustered using a graph-based shared nearest-neighbor clustering approach and plotted by a Uniform Manifold Approximation and Projection (UMAP) plot. The distribution of (A) total RNA molecules (nUMI) and (B) unique genes per cell (nGene) was visualized by UMAP plots.

Fig S4.

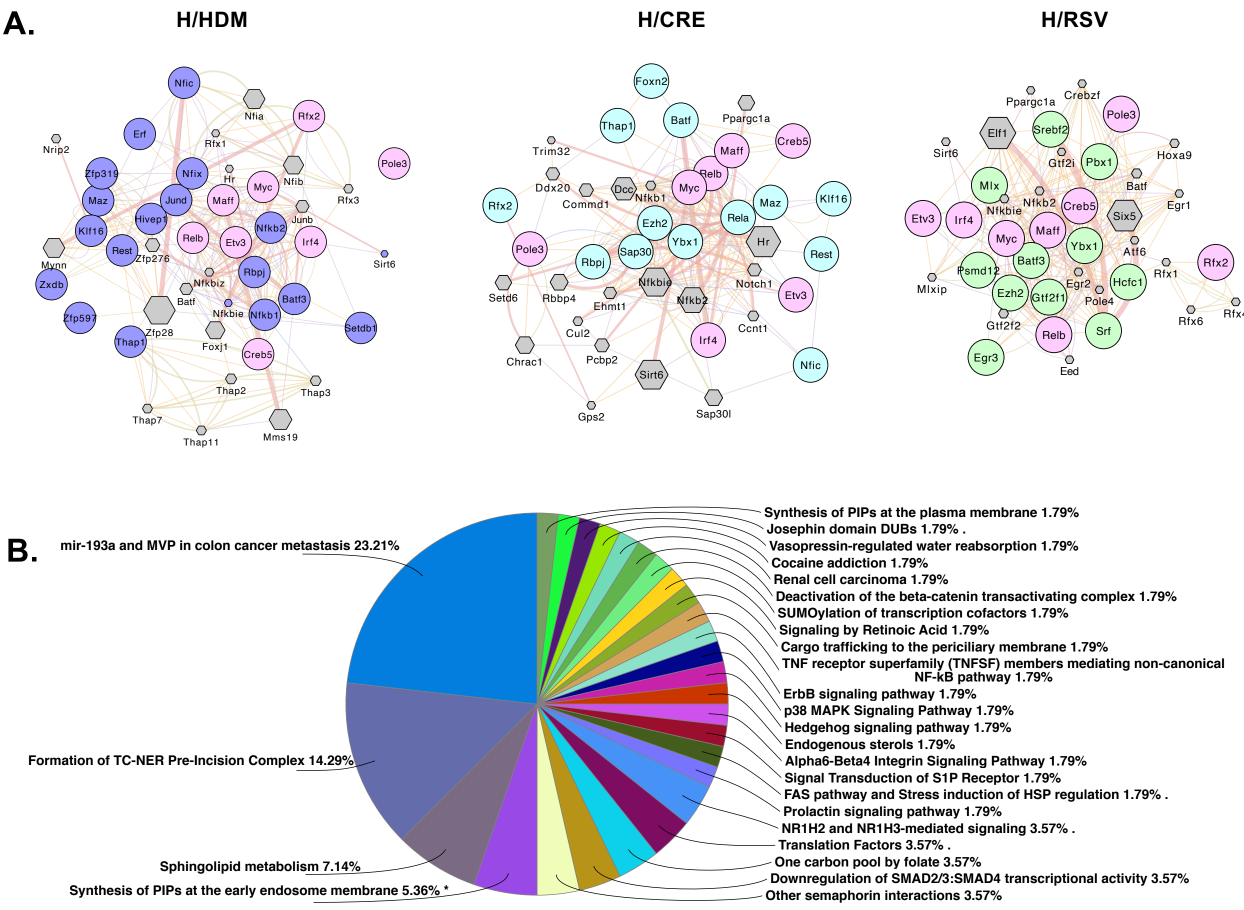

Supplementary Figure 5. SCENIC R package was employed to link the cis-regulatory sequence together with scRNA-seq data of H/HDM, H/CRE and H/RSV groups, and evaluated regulon activity scores for each single-cell reading. (A) Network interaction of regulons as identified by SCENIC analysis and plotted by Genemania Cytoscape package. Blue circles represent unique regulons for the H/HDM group, light blue circles for the H/CRE group, and green circles for the H/RSV group. Pink circles represent shared regulons for the aforementioned three groups. Grey pentagons represent transcriptional factors that were identified by in silico Genemania but not by SCENIC analysis. (B) Graphical representation of ClueGO analysis demonstrating the relative percentage of share signaling pathways employed by 166 overlapped genes shared between exacerbation groups.

Fig S5.

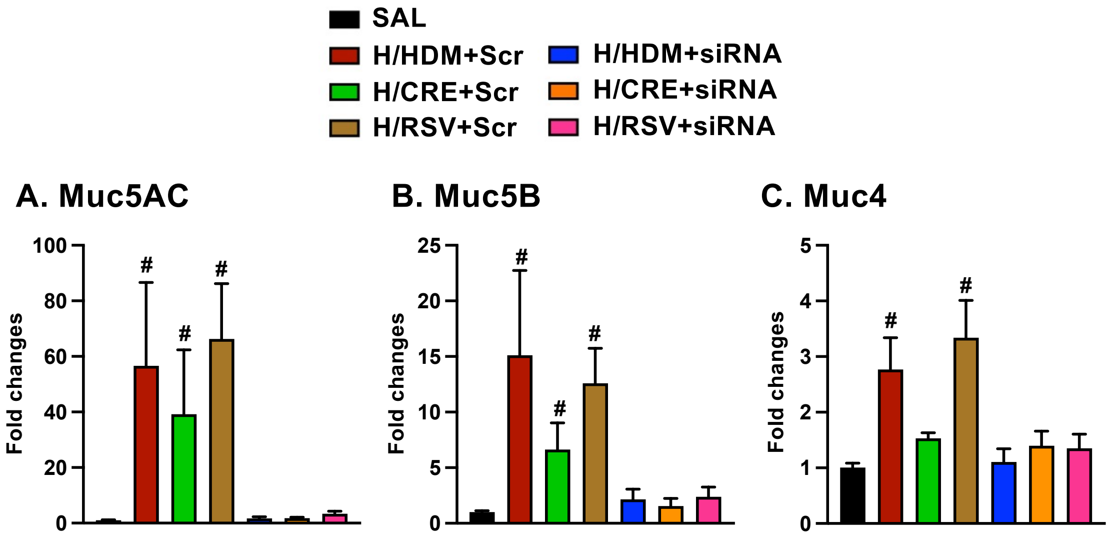

Supplementary Figure 6. Gene expression of MUC5AC (A), MUC5B (B), and MUC4 (C) relative to HPRT expression in lung tissue from exacerbated mice treated with Creb5 siRNA. ± SEM, n=4-6. #Designates significant differences compared to both SAL and siRNA treated groups (#p < 0.05).
